# Review of Clinical Applications of Dual-Energy CT in Patients after Endovascular Aortic Repair

**DOI:** 10.3390/jcm12247766

**Published:** 2023-12-18

**Authors:** Wojciech Kazimierczak, Natalia Kazimierczak, Zbigniew Serafin

**Affiliations:** 1 Collegium Medicum, Nicolaus Copernicus University in Torun, Jagiellońska 13-15, 85-067 Bydgoszcz, Poland; 2 Kazimierczak Private Medical Practice, Dworcowa 13/u6a, 85-009 Bydgoszcz, Poland

**Keywords:** dual-energy computed tomography, endoleaks, abdominal aortic aneurysm, virtual monoenergetic images, metal artifact reduction, diagnostic accuracy, computed tomography angiography

## Abstract

Abdominal aortic aneurysms (AAAs) are a significant cause of mortality in developed countries. Endovascular aneurysm repair (EVAR) is currently the leading treatment method for AAAs. Due to the high sensitivity and specificity of post-EVAR complication detection, CT angiography (CTA) is the reference method for imaging surveillance in patients after EVAR. Many studies have shown the advantages of dual-energy CT (DECT) over standard polyenergetic CTA in vascular applications. In this article, the authors briefly discuss the technical principles and summarize the current body of literature regarding dual-energy computed tomography angiography (DECTA) in patients after EVAR. The authors point out the most useful applications of DECTA in this group of patients and its advantages over conventional CTA. To conduct this review, a search was performed using the PubMed, Google Scholar, and Web of Science databases.

## 1. Introduction

Dual-energy CT (DECT) is a rapidly evolving diagnostic method first described by Sir Godfrey Hounsfield in 1973. He observed that dual image acquisition of the same volume at various kilovoltages allows for differentiation between calcium and iodine [1]. DECT enables the simultaneous or nearly simultaneous acquisition of CT images in low- and high-energy spectra, which allows for the differentiation of certain materials. The ability to differentiate elements using DECT stems from their distinct atomic numbers, unique k-edge characteristics, and differing linear attenuation coefficients at high and low photon energies. Some of the primary elements differentiated by DECT include iodine, which enables the creation of virtual noncontrast (VNC) and iodine map reconstructions. Other widely used reconstruction types in DECT are Virtual Monoenergetic Images (VMI), which simulate images acquired with a single-photon energy level.

Abdominal aortic aneurysms (AAAs) affect more than 1 million adults in the United States and result in approximately 15,000 annual deaths, making them the 15th leading cause of death overall [2,3,4]. Currently, the preferred treatment method for AAAs is endovascular aneurysm repair (EVAR), which carries the risk of a unique complication called an endoleak. This can lead to further aneurysm expansion and potential rupture, with a high mortality rate of 67% [5,6]. As a result, patients require regular imaging examinations to detect and classify endoleaks and identify other life-threatening complications, such as device thrombosis or infection. Various imaging modalities can be utilized during follow-up after EVAR; however, computed tomography angiography (CTA) and duplex ultrasound (DUS) are the basis for EVAR follow-up imaging [3] Despite the heterogeneous results of studies concerning the diagnostic accuracy of DUS [7,8] and its undeniable flaws (significant operator and patient dependencies), CTA is currently the reference standard imaging modality for post-EVAR patients. It allows for the classification of endoleaks and the detection of other potential complications [3,9].

The primary objective of this article was to elucidate the principles of DECTA, outline its advantages in post-EVAR patient follow-up imaging, and offer guidance for incorporating this technique into daily clinical practice.

## 2. Dual-Energy Acquisition Techniques

### 2.1. Rapid-Kilovoltage Switching DECT

**Figure 1:**
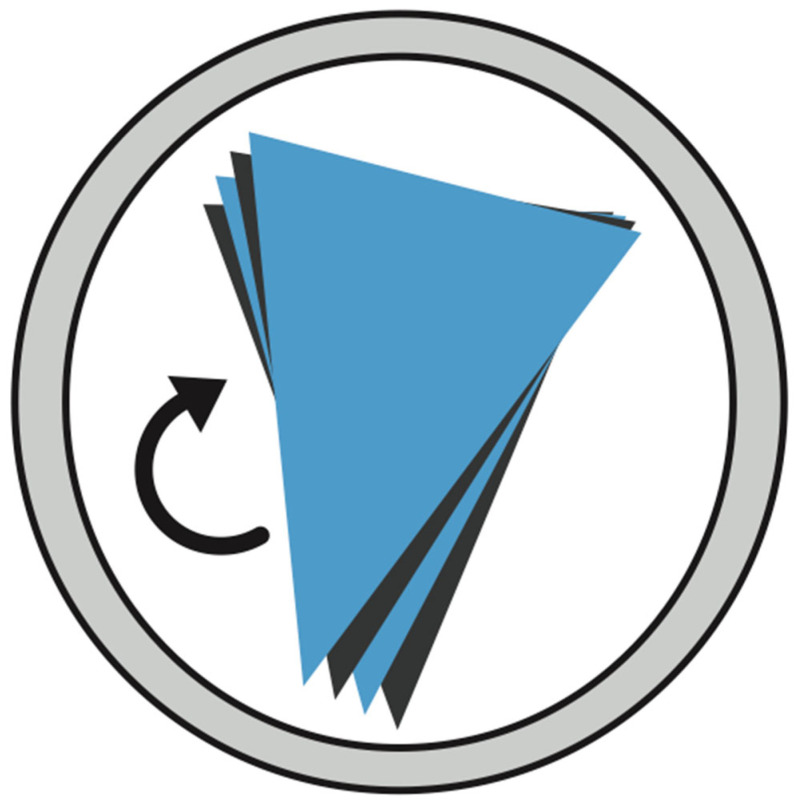
Schematic illustration of the single-source rapid kVp-switching DECT system (GE Healthcare). Dual-energy datasets are acquired by rapidly switching between low- and high-energy spectra. The system employs a unique garnet-based scintillator detector with minimal afterglow and quick sampling abilities.

The rapid-kVp (kilovoltage–peak) switching technique (Revolution GSI, Discovery 750 HD; General Electric Healthcare, Milwaukee, WI, USA) utilizes a single X-ray tube that quickly alternates (approximately every 0.25 ms) between 80 and 140 kVp, along with a single ultrafast registering detector, allowing for nearly simultaneous acquisition of two datasets. A schematic of the rapid kVp switching system is shown in Figure 1.

### 2.2. Dual-Source DECT

**Figure 2:**
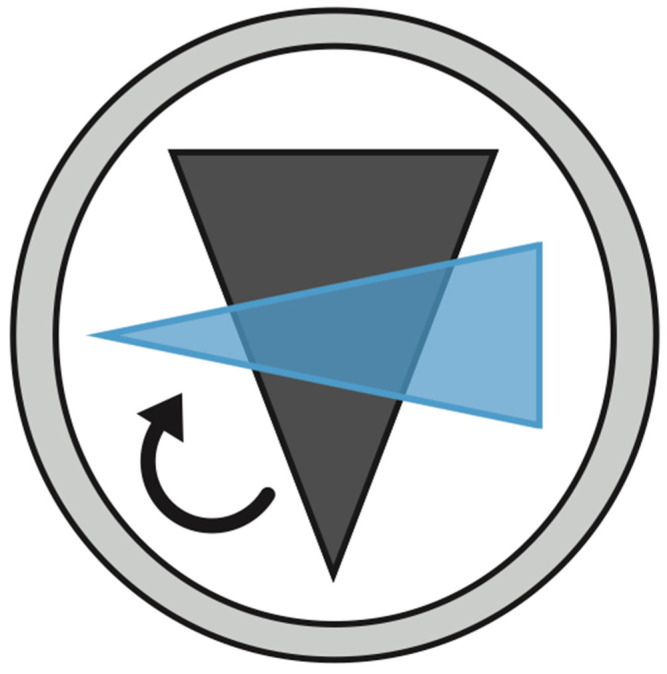
Schematic of the dual-source DECT system (Siemens AG). The system utilizes two dual-source detector–scanner combinations in a nearly orthogonal configuration, allowing for simultaneous volume scanning at the two energies. Typically, the sources operate at 80–100 kVp and 140–150 kVp; other combinations may be used for specific applications. Additional filters that can be used to harden a high-energy beam may be used to achieve better spectral separation. The limited space in the CT gantry, which allows for a smaller second detector, restricts the usable field of view.

A dual-source, dual-energy CT scanner (Somatom Definition Flash, Somatom Force; Siemens Healthineers, Forchheim, Germany) utilizes two sets of separate detector rings and two X-ray tubes positioned at 90° around the CT gantry. The X-ray tubes operate at low (70–80 kVp) and high (140–150 kVp) energies independently, allowing for the simultaneous acquisition of two datasets. A schematic of the dual-source system is shown in Figure 2.

### 2.3. Split-Filter DECT

**Figure 3:**
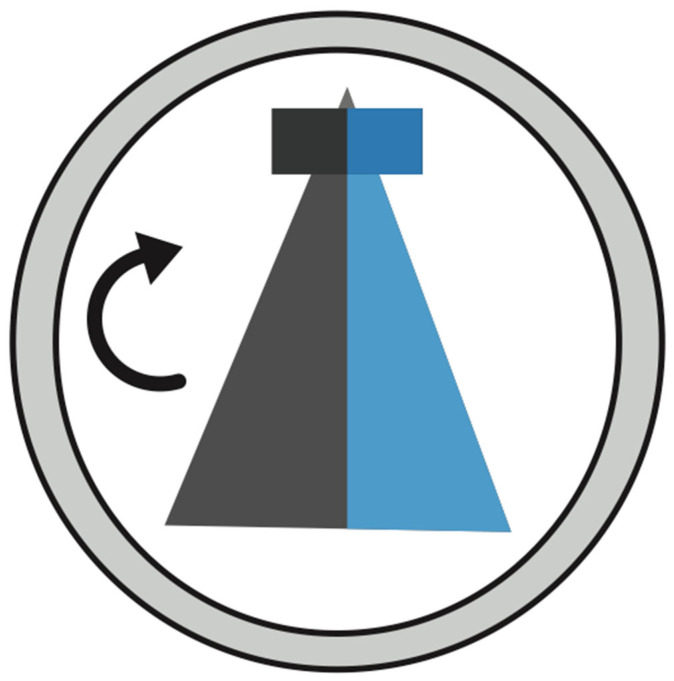
Schematic illustration of the **s**ingle-source split-filter DECT system (Siemens AG). The filter, which was divided into two parts composed of gold and tin, was positioned at the output of the tube. This causes the beam to separate into low- and high-energy spectra. The respective halves of the detector then facilitate the acquisition of dual-energy datasets.

The split-filter system (Somatom Definition Edge and Somatom go.Top; Siemens Healthineers) allows for the simultaneous acquisition of high- and low-energy datasets. In this system, a 120 kVp X-ray beam is prefiltered with gold and tin filters, splitting the beam energy into two spectra before it reaches the patient. The scheme of the split-filter system is shown in Figure 3.

### 2.4. Multilayer Detector CT

**Figure 4:**
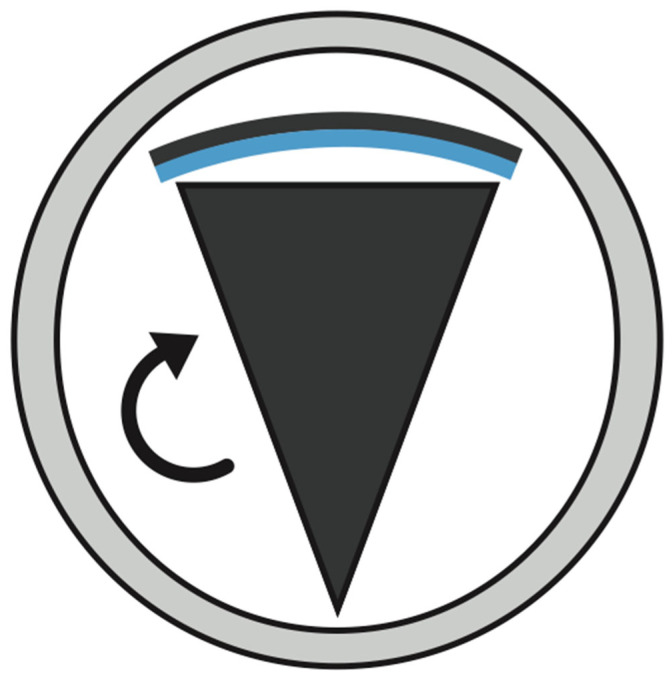
Schematic illustration of the single-source layered detector DECT system (Phillips Healthcare). The dual-energy datasets are achieved via spectral separation at the detector level. This system capitalizes on the polychromatic beam generated at the source and employs specialized dual-layer detectors sensitive to a specific energy spectrum. The superficial layer, which absorbs approximately 50% of the total photons, is designed to primarily absorb low-energy photons. The second layer absorbs the remaining high-energy photons.

The dual-layer detector (IQon Spectral CT; Philips Medical Systems, Cleveland, OH, USA) employs a layered or “sandwich” scintillation detector, which allows for the simultaneous collection of two datasets from a single standard X-ray tube operating at 140 kVp. The low-energy data are obtained from the top yttrium-based layer, while the high-energy data are collected from the bottom, a gadolinium-oxysulfide-based layer. A diagram of the multilayer detector is shown in Figure 4.

## 3. Dual-Energy CT Postprocessing Techniques

### 3.1. Material Decomposition

Material-specific information can be obtained by modeling attenuation profiles, material mass density, and atomic number (Z) maps [10,11]. This allows for the reconstruction of images coded with concentrations of certain elements and substances instead of the simple CT attenuation numbers of each voxel in conventional single-energy CT (SECT). Material decomposition facilitates the precise mapping of specific elements, thereby enabling additional reconstructions, such as the virtual subtraction of elements. In vascular studies, the most beneficial images are those from VNC and virtual noncalcium (VNCa) reconstructions.

### 3.2. Virtual Noncontrast and Iodine Mapping

**Figure 5:**
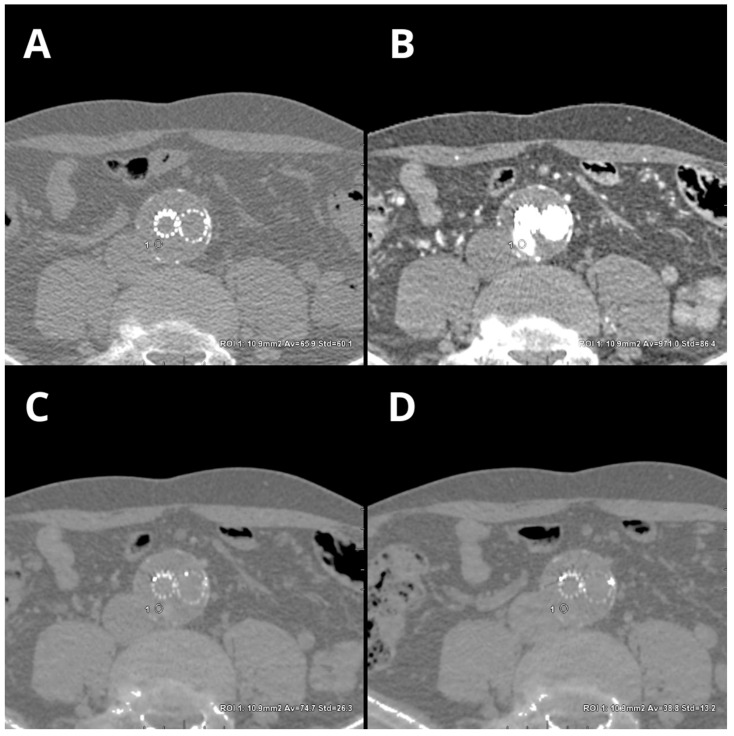
Differences in CT attenuation (average density ± SD) in small endoleak cases with true non-contrast, 40 keV VMI (arterial phase), and two VNC phases. Reconstructions: TNC ((**A**)—65.9 ± 60.1 HU), 40 keV VMI ((**B**)—971 ± 86.4), VNC arterial ((**C**)—74.7 ± 26.3 HU), and VNC delayed ((**D**)—38.8 ± 13.2 HU). An automatic region-of-interest (ROI) propagation tool was used with the same window settings (W 500, L 100).

Dual-energy CT has the potential to generate VNC images via iodine identification and subsequent subtraction. These VNC images mimic the appearance of true noncontrast (TNC) images. Numerous studies in various clinical settings have proven that VNC images can substitute for TNC images and that the TNC phase of multiphasic examinations can be omitted [12,13,14,15,16]. VNC images have been proven to be vulnerable to iodine content, leading to significant differences in CT numbers between TNC and VNC images derived from arterial and delayed examination phases [17,18]. Figure 5 shows the differences between the TNC and the two postcontrast VNC reconstructions.

Another benefit of material decomposition and VNC phase reconstruction is the ability to create iodine maps, which represent the distribution of iodine in tissues [19]. The next step is color coding of iodine, highlighting the iodine content in the grayscale VNC images. Such reconstructions are particularly useful in oncological applications and endoleak detection [20,21,22].

### 3.3. Virtual Noncalcium

VNCa algorithms facilitate the removal of calcified plaques without affecting intraluminal iodine-based contrast agent and surrounding soft tissues [23]. VNCa algorithms are particularly useful for imaging narrow vessel stenosis caused by calcified plaques. Furthermore, VNCa algorithms allow for the reduction of streaks and beam-hardening artifacts that obscure the lumen of the vessel and surrounding soft tissues [23,24]. The VNCa algorithm has already proven its value in assessing carotid artery stenosis, reducing blooming artifacts, and mitigating the overestimation of stenosis [23].

### 3.4. Virtual Monoenergetic Images (VMI), Noise Optimization

Virtual monoenergetic images (VMIs) can be reconstructed from dual-energy CT acquisitions, mimicking the attenuation values of an image obtained using a single energy source. Generally, low-keV images (40–70 keV) are advantageous for increasing iodine contrast but can also lead to higher noise levels [25]. Low-keV datasets can be particularly useful for improving iodine contrast, such as in low contrast volumes at slow injection rates [26], improving the detection and delineation of poorly enhancing lesions [27,28], assessing coronary vasculature, and performing functional evaluation of the myocardium [29,30].

Grant et al. introduced enhancements to the VMI technique to address the challenge of image noise and improve the iodine contrast-to-noise ratio (CNR)-optimized virtual monoenergetic image (VMI+) reconstructions [31]. In addition to noise reduction, the VMI+ technique mitigates artifacts, such as those from beam hardening and photon starvation, that may arise from high-attenuation materials at higher energy levels [32,33].

## 4. Applications of DECT in Patients after EVAR

### 4.1. Radiation Dose Reduction

The ionizing radiation dose associated with lifelong diagnostic surveillance is a fundamental problem related to post-EVAR follow-up protocols. The risk of radiation-induced cancer related to repeated CT scans is already well established [34,35,36,37,38]. The basic methods of radiation dose reduction are automatic exposure systems, iterative techniques, and regular service of the tomographic device [39]. One way to reduce the radiation dose is to lower the tube current and voltage; however, this increases the image noise and decreases the diagnostic value of the examination [40].

Among the concerns regarding dual-energy CT examinations, the most significant and recurring are those related to radiation dose. Several studies have demonstrated that the dose delivered during dual-source dual-energy CT acquisition is similar to that of comparable SECT [41,42,43,44]. With the advancement of technology, the introduction of iterative techniques, improved detector efficiency, and spectral filtration systems have made it possible to deliver even lower radiation doses with DECT than with SECT [26,45].

Several researchers have highlighted the possibility of using shortened examinations, limited to phases performed after administering a contrast agent, with VNC phase reconstruction without a significant reduction in the sensitivity of CTA for detecting endoleaks [46,47,48,49,50,51]. The dose reduction obtained in these studies primarily results from skipping the native phase of the examination. The aforementioned studies also pointed out the possibility of dose reduction by additionally skipping the arterial phase of the examination while maintaining the high sensitivity of the single-phase protocol for detecting endoleaks. A summary of these studies is provided in Table 1.

Since cumulative radiation exposure is a fundamental factor influencing post-EVAR diagnostic surveillance protocols, CTA protocols are a compromise between radiation dose and diagnostic accuracy. There is generally a consensus among researchers regarding reducing the number of examination phases by omitting the native phase and reconstructing the VNC. However, the number of examination phases performed after administering a contrast agent is controversial. A few authors have highlighted the importance of the arterial phase in detecting endoleaks, demonstrating a higher sensitivity of multiphasic (VNC + 2 postcontrast DECT acquisitions) examination protocols [48,51]. Potentially life-threatening and requiring treatment, type I and type III endoleaks require the arterial phase of examination for diagnosis [52]. Despite discrepancies in the literature on the necessity of the arterial phase for detecting endoleaks, this phase unquestionably holds value in assessing the potential narrowing of abdominal arteries [53,54]. This issue is particularly significant in the case of post-br/fEVAR procedures due to a higher risk of complications within the target arteries. Furthermore, the arterial phase allows for the evaluation of perfusion disorders of the abdominal organs and potentially the implementation of appropriate treatment. In the general elderly population of EVAR patients, acquiring the arterial phase might be particularly important in assessing additional findings such as tumors. Therefore, the presence of the arterial phase in CTA protocols appears justified. However, the optimal scanning protocol for CT scanning remains controversial [48,49,51,55,56,57].

An interesting study in this context was conducted by Javor et al., who demonstrated the possibility of reducing the radiation dose by 42% via a split-bolus technique with one DE acquisition and VNC reconstruction with 96% sensitivity in endoleak detection [58]. Similar results were achieved by Boos and Iezzi [59,60]. In theory, this technique allows for the optimal contrast of low- and high-flow endoleaks, as well as arterial vessels and parenchymal organs, during a single scan. However, despite promising results, the literature lacks sufficient evidence for the effectiveness of split-bolus protocols in post-EVAR surveillance.

### 4.2. Contrast Agent Volume Reduction

The use of contrast agents is mandatory in both procedure planning and post-EVAR diagnostic surveillance. The accuracy of delineating three-dimensional vessel structures is crucial for the selection of proper surgical devices and the diagnosis of postprocedural complications. However, high-quality CTA requires the administration of an appropriate volume of iodine contrast agent. The use of contrast agents is associated with adverse effects, such as hypersensitivity allergic reactions, thyroid dysfunction, and nephropathy [61,62,63]. To minimize the risk of contrast media-induced nephropathy, it is recommended to use as o.w. volume of a contrast agent as possible for diagnostic imaging [64,65,66]. Therefore, contrast agent volume reduction techniques are used.

The phenomenon of high CT attenuation numbers in low-level virtual monoenergetic images (VMIs) is well established [25,67]. When low-level VMIs are used, CT attenuation of the contrast material can be increased, allowing for the injection of a lower dose of iodine [68]. Low-level VMIs have been shown to boost vascular contrast in several vascular beds, which can reduce the volume of the contrast agent [69,70,71]. Studies have shown that necessary preprocedural measurements of the aorta can be acquired with low-level VMIs, permitting imaging with an equivalent radiation dose but a lower contrast dose than standard SECT [72,73].

**Figure 6:**
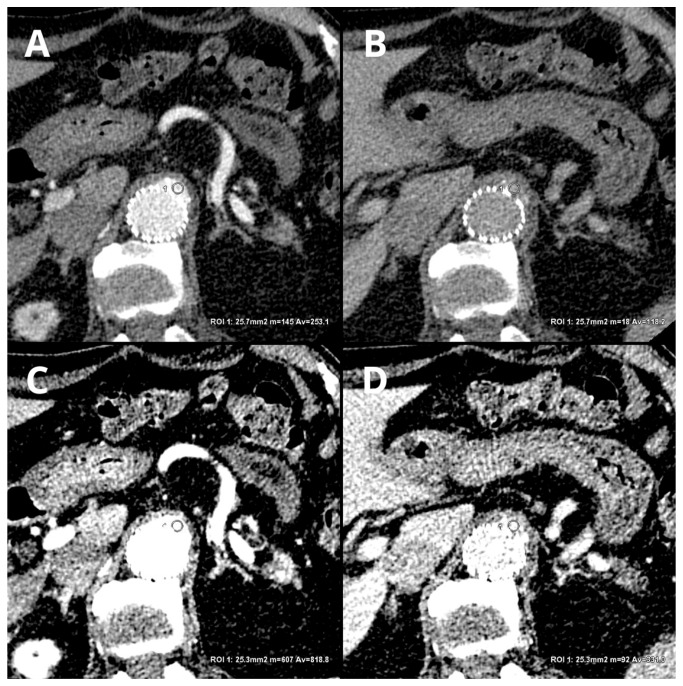
Comparison of LB and 40 keV VMI reconstructions in arterial and delayed phases: LB arterial ((**A**)—253.1 ± 50.2 HU), LB delayed ((**B**)—118.2 ± 25.4), 40 keV VMI arterial ((**C**)—818.8 ± 116.5 HU), and 40 keV VMI delayed ((**D**)—331 ± 53.4 HU). An automatic region-of-interest (ROI) propagation tool was used. The same window settings (W 500, L 100) were used to highlight the differences in the contrast visualization. LB-delayed and 40 keV VMI-delayed images serve as examples of the potential to salvage an examination with a reduced volume of contrast agent.

Currently, there is a lack of data in the literature on the diagnostic accuracy of protocols involving reduced contrast agent administration. Despite this, this issue is of significant importance in the context of follow-up CTA of EVAR patients. In clinical practice, there are instances of administering reduced amounts of contrast agents due to staff errors, disconnection of injecting system components, access vessel rupture, or incorrect acquisition timing. Additionally, radiological protection concerns, such as avoiding the repetition of poorly performed examinations, justify the need to implement methods that allow for the assessment of CTA with suboptimal vessel enhancement. Low-energy VMIs may enable a reduction in rejected examinations and provide a reliable assessment of endoleaks in these specific clinical settings. Figure 6 shows the differences between conventional, linearly blended, and low-level VMI reconstructions in type 3 endoleaks.

### 4.3. Endoleak Detection

Low-level VMIs are a major factor in the superiority of DECT over SECT for detecting endoleaks. To date, few studies have assessed the impact of low-level VMIs on the diagnostic accuracy of endoleak detection [51,74,75,76]. A study by Maturen et al. [74] showed a higher sensitivity for endoleak detection with a VMI of 55 keV than a VMI of 75 keV. Martin et al. [75] reported a significantly greater rate of endoleak detection in VMI and VMI+ compared to standard linearly blended images (LB). These results were accompanied by a significant improvement in the image quality parameters (contrast-to-noise ratio). Comparable results were achieved by Kazimierczak et al. [51] in a 2023 study, showing a significant increase in the number of endoleaks diagnosed (an increase of almost 30%) and an improvement in image quality parameters with 40 keV VMI compared to LB images. Charalombous et al. reported the use of a 54 keV VMI to enhance the efficiency of endoleak detection efficiency. Moreover, analysis of the normalized effective atomic number and improvised endoleak index was found to have significant power in predicting the aggressiveness of type II endoleaks [77]. However, all of the mentioned studies were conducted on relatively small study groups with fewer than 100 patients and did not influence the current guidelines regarding post-EVAR follow-up. An interesting study by Skawran et al. [76] compared low-level VMIs and single-energy low-kV images (SEIs) in terms of the diagnostic accuracy of six readers in endoleak detection as well as subjective and objective image quality properties. The results of this study indicated that a low-keV VMI+ improved the contrast-to-noise ratio of the aorta. However, the noise level, subjective image quality, and diagnostic accuracy of endoleaks were superior for SEI. Although the results of the present study are related to analyses performed on a phantom, they suggest a promising direction for further research to improve the detectability of endoleaks.

### 4.4. Metal Artifact Reduction

Materials used in vascular procedures, such as coils, embolization materials, and stent graft materials, can cause artifacts in CT scans, primarily photon starvation and beam hardening artifacts. These artifacts can hamper image quality and significantly decrease the diagnostic value of the examination. Metal artifact reduction (MAR) algorithms theoretically find particular applications in patients after br/fEVAR.

The presence of metallic markers on fenestrations, branches, stents, the metal structure of stent grafts, and previously used coils or embolization materials can result in artifacts that lower the diagnostic value of the examination. Beam-hardening artifacts make it difficult to assess stented vessels, preventing proper evaluation of potential narrowing or occlusion. In the case of significant artifacts, vessel patency assessment must rely on the evaluation of potential collateral circulation, contrast enhancement of the distal branches of the evaluated vessel, and the presence of hypoperfusion/infarction signs in the organ supplied by the studied artery. Early detection of narrowing may allow the implementation of treatment to prevent the development of complete occlusion and, consequently, organ infarction [78]. Additionally, metallic artifacts can mask the presence of small endoleaks in patients after EVAR.

Theoretically, using MAR algorithms can improve the diagnostic value of patients after EVAR [56,59]. However, some studies indicate a significant decrease in the diagnostic value of MAR algorithms compared to that of DECT in a group of patients post-EVAR. In a study by Boos et al., the researchers aimed to assess the effectiveness of MAR algorithms in fast kV-switching DECTA in a group of 24 post-EVAR patients [59]. The primary objective was to determine whether the MAR technique could improve endoleak visualization and reduce the artifacts caused by the metallic components of EVAR stents and coils. The results of artifact evaluation showed an objective decrease in artifacts from EVAR stents in the near field, albeit associated with a subjective increase in artifacts in the near field, far field, and vessels. Furthermore, the MAR algorithm impaired visualization in 60% (n = 6) of patients with endoleaks and improved visualization in 10% (n = 1) of patient with endoleaks. In a recent study, MAR algorithms objectively improved visualization of stents in target vessels in patients after br/fEVAR but surprisingly significantly impaired subjective image quality (rate of 1.57 ± 0.5 on a 5-point Likert scale compared to a mean rate of 4.25 ± 0.44 for adaptive statistical iterative reconstructions) [79]. Additionally, the authors reported hampered endoleak visualization and additional artifacts that could result in false positive diagnoses of endoleaks. However, this topic requires further analysis because the results conflict with a substantial portion of the literature, as well as the very small study groups involved.

**Figure 7:**
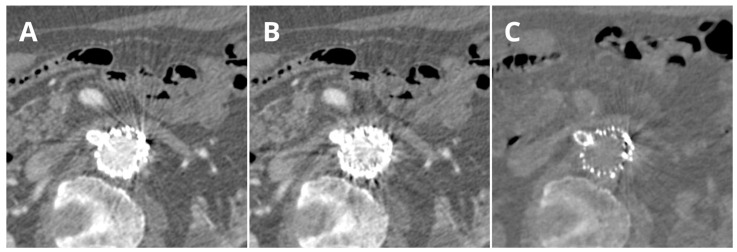
Comparison of the LB (**A**), MAR (**B**), and 140 keV VMI (**C**) reconstructions. Arterial phase: The level of the right renal artery (RRA) in a patient one month after the fEVAR procedure. The same window settings were used (W 500, L 100). It is important to note the additional artifacts in MAR reconstructions that completely prevent the evaluation of the initial segment of the stent to the RRA and the decreased contrast visualization on the 140 keV VMI. Artifact intensity variations between the reconstructions can be observed.

The solution to this problem appears to be to use high-keV VMI reconstructions (≥100 keV), which reduce blooming artifacts caused by hyperdense structures, such as calcified plaques and metal stents. This approach has proven particularly useful in cardiac CT scans [80]. Reconstructions in the range of 130–150 keV provide optimal imaging of stent lumens less than 3 mm in diameter, potentially reducing the dose of ionizing radiation [81,82]. Furthermore, these reconstructions enhance the diagnostic value of examinations plagued with artifacts associated with calcified plaques and an influx of contrast material [83,84]. A comparison of the MAR and 140 keV reconstructions is shown in Figure 7.

## 5. Limitations

Despite the numerous advantages of DECT in vascular imaging, concerns have been raised regarding workflow, artifacts, temporal misregistration, radiation exposure, and image quality [85]. A factor that directly affects the utility and frequency of DECT use is its integration into the workflow. Dual-energy CT imaging has been associated with multiple workflow issues, among which the most significant are increased reconstruction time, a large number of images (resulting in increased PACS usage and longer downloading times), and increased interpretation time [86,87]. DECT postprocessing requires exclusive vendor-specific software, which can be costly and vary in capabilities. Postprocessing of spectral data can be very time-consuming for both technologists and radiologists, and additional postprocessing may be impossible without the use of a full spectral dataset [88]. Generally, implementing DECT in a routine workflow requires substantial knowledge of the vendor’s scanner and results in a steep learning curve [89]. Moreover, DECT systems are associated with higher costs for specific hardware and software [85].

DECT imaging is susceptible to various artifacts related to scanner design, acquisition protocols, and postprocessing techniques, which may be unique to the platform utilized [85,90]. Image noise can be increased using certain reconstruction approaches (such as low-level VMIs). Patient size, motion, and iodine concentration also contribute to artifacts, potentially leading to nondiagnostic images. Incorrect attenuation thresholds may lead to false positive or false negative results in material decomposition protocols [90]. Additionally, some types of scanners (split-filter scanners) are characterized by a lower temporal resolution [90,91]. Moreover, increased body size can lead to greater image noise and lower quality in DECT abdominal imaging due to reduced photon detection and exacerbated beam-hardening artifacts [85]. Consequently, patient selection criteria based on weight and body dimensions have been suggested [90,92].

Initial concerns regarding the radiation dose combined with DECT have been mitigated by recent advances in technology [88]. Several studies have shown doses comparable to or lower than the delivered SECT radiation doses without compromising image quality [42,43,44,93,94]. Several techniques leading to significant radiation dose reduction, including VNC imaging, noise reduction algorithms, and limiting the FOV to the area of interest, have been utilized [95,96,97,98]. However, radiation doses can vary depending on the scanner model, scan type, body region, and patient factors [88]. Therefore, implementing DECT protocols requires staff to have specialized knowledge that allows for the efficient and safe use of this technology.

## 6. Conclusions

DECT is an emerging technology that offers additional layers of information inaccessible using conventional CT. DECT enables molecular composition analysis, opening new horizons in imaging that significantly surpasses standard tomographic examinations. With the increasing number of diverse dual-energy systems and their growing availability, we observed a steady increase in the applications of these technologies in various clinical settings. An increasing number of publications demonstrate the significant advantages of DECT over SECT, particularly in angiographic studies.

The application of spectral CT systems in patients after EVAR enhances the diagnostic value of these examinations. The most useful reconstructions were those obtained using material decomposition and VMI reconstruction. Because of the virtual nonenhanced phases, spectral CT angiography can be performed in EVAR patients with a significantly lower effective radiation dose and a potentially reduced contrast agent dosage. VMI reconstruction enhances the visualization of endoleaks and may assist in evaluating images marked by metal artifacts. The benefits of DECT in post-EVAR examinations are summarized in Table 2.

In summary, the implementation of DECT in patients after EVAR allows for a reduction in ionizing radiation dose and an increase in the diagnostic value of the examination in detecting postprocedural complications. However, implementing DECT acquisitions in clinical practice remains a challenge.

## Figures and Tables

**Table 1:** Reduction in the average radiation dose in DECT studies compared with the triphasic examination protocol.

Research	Protocol		Dose Reduction (%)
	Mono-Phasic (mSv)	Three-Phasic (mSv)	
Chandarana et al., 2008 [47]	11.1	27.8	61
Flors et al., 2013 [48]	9.8	22.4	64.1
Stolzman et al., 2008 [49]	10.9	27.4	61
Buffa et al., 2014 [50]	10.5	27.4	61.7
Kazimierczak et al., 2023 [51]	10.69	27.96	61.37

**Table 2:** Summary of the advantages of DECT over conventional SECT and its clinical applications.

Reconstruction Technique	Advantage	Application
Low-energy VMI	Higher sensitivity for iodine.	Improved endoleak detection. Contrast dose reduction. Salvage of suboptimal contrast examination.
High-energy VMI	Reduction in calcium blooming artifacts. Metal artifact and beam-hardening reduction.	Reduction in artifacts from stentgraft structures and embolization materials. Better visualization of stent lumen. Improved visualization of calcified vessels.
Virtual noncontrast images	Reduction in number of phases of examination.	Reduction in radiation dose. Characteristic of incidental findings in abbreviated examination protocols (without true noncontrast phase).
Material decomposition	Identification of elemental composition of tissues.	Plaque characterization. Improved separation of calcium from iodine.

## Data Availability

Not applicable.

## References

[1] Hounsfield G.N. (1973). Computerized transverse axial scanning (tomography): I. Description of system. Br. J. Radiol..

[2] Stather P., Sidloff D., Rhema I., Choke E., Bown M., Sayers R. (2014). A review of current reporting of abdominal aortic aneurysm mortality and prevalence in the literature. Eur. J. Vasc. Endovasc. Surg..

[3] Chaikof E.L., Dalman R.L., Eskandari M.K., Jackson B.M., Lee W.A., Mansour M.A., Mastracci T.M., Mell M., Murad M.H., Nguyen L.L. (2018). The Society for Vascular Surgery practice guidelines on the care of patients with an abdominal aortic aneurysm. J. Vasc. Surg..

[4] Lederle F.A., Johnson G.R., Wilson S.E., Chute E.P., Littooy F.N., Bandyk D., Krupski W.C., Barone G.W., Acher C.W., Ballard D.J. (1997). Prevalence and associations of abdominal aortic aneurysm detected through screening. Ann. Intern. Med..

[5] Schlösser F., Gusberg R., Dardik A., Lin P., Verhagen H., Moll F., Muhs B. (2009). Aneurysm Rupture after EVAR: Can the Ultimate Failure be Predicted?. Eur. J. Vasc. Endovasc. Surg..

[6] Schermerhorn M.L., Buck D.B., O’malley A.J., Curran T., McCallum J.C., Darling J., Landon B.E. (2015). Long-Term Outcomes of Abdominal Aortic Aneurysm in the Medicare Population. N. Engl. J. Med..

[7] Karaolanis G.I., Antonopoulos C.N., Georgakarakos E., Lianos G.D., Mitsis M., Glantzounis G.K., Giannoukas A., Kouvelos G. (2022). Colour Duplex and/or Contrast-Enhanced Ultrasound Compared with Computed Tomography Angiography for Endoleak Detection after Endovascular Abdominal Aortic Aneurysm Repair: A Systematic Review and Meta-Analysis. J. Clin. Med..

[8] Mirza T., Karthikesalingam A., Jackson D., Walsh S., Holt P., Hayes P., Boyle J. (2010). Duplex Ultrasound and Contrast-Enhanced Ultrasound Versus Computed Tomography for the Detection of Endoleak after EVAR: Systematic Review and Bivariate Meta-Analysis. Eur. J. Vasc. Endovasc. Surg..

[9] Wanhainen A., Verzini F., Van Herzeele I., Allaire E., Bown M., Cohnert T., Dick F., van Herwaarden J., Karkos C., Koelemay M. (2019). Editor’s Choice—European Society for Vascular Surgery (ESVS) 2019 Clinical Practice Guidelines on the Management of Abdominal Aorto-iliac Artery Aneurysms. Eur. J. Vasc. Endovasc. Surg..

[10] Alvarez R.E., MacOvski A. (1976). Energy-selective reconstructions in X-ray computerised tomography. Phys. Med. Biol..

[11] McCollough C.H., Leng S., Yu L., Fletcher J.G. (2015). Dual- and multi-energy CT: Principles, technical approaches, and clinical applications. Radiology.

[12] Connolly M.J., McInnes M.D.F., El-Khodary M., McGrath T.A., Schieda N. (2017). Diagnostic accuracy of virtual non-contrast enhanced dual-energy CT for diagnosis of adrenal adenoma: A systematic review and meta-analysis. Eur. Radiol..

[13] Lehti L., Söderberg M., Höglund P., Nyman U., Gottsäter A., Wassélius J. (2018). Reliability of virtual non-contrast computed tomography angiography: Comparing it with the real deal. Acta Radiol. Open.

[14] Takahashi N., Hartman R.P., Vrtiska T.J., Kawashima A., Primak A.N., Dzyubak O.P., Mandrekar J.N., Fletcher J.G., McCollough C.H. (2008). Dual-energy CT iodine-subtraction virtual unenhanced technique to detect urinary stones in an iodine-filled collecting system: A phantom study. Am. J. Roentgenol..

[15] Graser A., Johnson T.R.C., Hecht E.M., Becker C.R., Leidecker C., Staehler M., Stief C.G., Hildebrandt H., Godoy M.C.B., Finn M.E. (2009). Dual-energy CT in patients suspected of having renal masses: Can virtual nonenhanced images replace true nonenhanced images?. Radiology.

[16] Phan C., Yoo A., Hirsch J., Nogueira R., Gupta R. (2012). Differentiation of hemorrhage from iodinated contrast in different intracranial compartments using dual-energy head CT. Am. J. Neuroradiol..

[17] Lehti L., Söderberg M., Höglund P., Wassélius J. (2019). Comparing Arterial- and Venous-Phase Acquisition for Optimization of Virtual Noncontrast Images from Dual-Energy Computed Tomography Angiography. J. Comput. Assist. Tomogr..

[18] Kazimierczak W., Kazimierczak N., Serafin Z. (2023). Quality of virtual-non-contrast phases derived from arterial and delayed phases of fast-kVp switching dual-energy CT in patients after endovascular aortic repair. Int. J. Cardiovasc. Imaging.

[19] Heye T., Nelson R.C., Ho L.M., Marin D., Boll D.T. (2012). Dual-energy CT applications in the abdomen. AJR Am. J. Roentgenol..

[20] Virarkar M.K., Vulasala S.S.R., Gupta A.V., Gopireddy D., Kumar S., Hernandez M., Lall C., Bhosale P. (2022). Virtual Non-contrast Imaging in The Abdomen and The Pelvis: An Overview. Seminars in Ultrasound, CT and MRI.

[21] Ascenti G., Sofia C., Mazziotti S., Silipigni S., D’Angelo T., Pergolizzi S., Scribano E. (2016). Dual-energy CT with iodine quantification in distinguishing between bland and neoplastic portal vein thrombosis in patients with hepatocellular carcinoma. Clin. Radiol..

[22] Ascenti G., Mazziotti S., Lamberto S., Bottari A., Caloggero S., Racchiusa S., Mileto A., Scribano E. (2011). Dual-energy CT for detection of endoleaks after endovascular abdominal aneurysm repair: Usefulness of colored iodine overlay. Am. J. Roentgenol..

[23] Mannil M., Ramachandran J., de Martini I.V., Wegener S., Schmidt B., Flohr T., Krauss B., Valavanis A., Alkadhi H., Winklhofer S. (2017). Modified Dual-Energy Algorithm for Calcified Plaque Removal: Evaluation in Carotid Computed Tomography Angiography and Comparison with Digital Subtraction Angiography. Investig. Radiol..

[24] Silvennoinen H., Ikonen S., Soinne L., Railo M., Valanne L. (2007). CT angiographic analysis of carotid artery stenosis: Comparison of manual assessment, semiautomatic vessel analysis, and digital subtraction angiography. Am. J. Neuroradiol..

[25] Hu D., Yu T., Duan X., Peng Y., Zhai R. (2014). Determination of the optimal energy level in spectral CT imaging for displaying abdominal vessels in pediatric patients. Eur. J. Radiol..

[26] Siegel M.J., Ramirez-Giraldo J.C. (2019). Dual-energy CT in children: Imaging algorithms and clinical applications. Radiology.

[27] De Cecco C.N., Caruso D., Schoepf U.J., De Santis D., Muscogiuri G., Albrecht M.H., Meinel F.G., Wichmann J.L., Burchett P.F., Varga-Szemes A. (2018). A noise-optimized virtual monoenergetic reconstruction algorithm improves the diagnostic accuracy of late hepatic arterial phase dual-energy CT for the detection of hypervascular liver lesions. Eur. Radiol..

[28] Zhang X., Zhang G., Xu L., Bai X., Lu X., Yu S., Sun H., Jin Z. (2022). Utilisation of virtual non-contrast images and virtual mono-energetic images acquired from dual-layer spectral CT for renal cell carcinoma: Image quality and radiation dose. Insights Imaging.

[29] Ko S.M., Choi J.W., Song M.G., Shin J.K., Chee H.K., Chung H.W., Kim D.H. (2010). Myocardial perfusion imaging using adenosine-induced stress dual-energy computed tomography of the heart: Comparison with cardiac magnetic resonance imaging and conventional coronary angiography. Eur. Radiol..

[30] Wichmann J.L., Bauer R.W., Doss M., Stock W., Lehnert T., Bodelle B., Frellesen C., Vogl T.J., Kerl J.M. (2013). Diagnostic accuracy of late iodine-enhancement dual-energy computed tomography for the detection of chronic myocardial infarction compared with late gadolinium-enhancement 3-T magnetic resonance imaging. Investig. Radiol..

[31] Grant K.L., Flohr T.G., Krauss B., Sedlmair M., Thomas C., Schmidt B. (2014). Assessment of an Advanced Image-Based Technique to Calculate Virtual Monoenergetic Computed Tomographic Images from a Dual-Energy Examination to Improve Contrast-To-Noise Ratio in Examinations Using Iodinated Contrast Media. Investig. Radiol..

[32] Zeng Y., Geng D., Zhang J. (2021). Noise-optimized virtual monoenergetic imaging technology of the third-generation dual-source computed tomography and its clinical applications. Quant. Imaging Med. Surg..

[33] Wichmann J.L., Gillott M.R., De Cecco C.N., Mangold S., Varga-Szemes A., Yamada R., Otani K., Canstein C.M., Fuller S.R.B., Vogl T.J. (2016). Dual-Energy Computed Tomography Angiography of the Lower Extremity Runoff. Investig. Radiol..

[34] Dixon A.K., Dendy P. (1998). Spiral CT: How much does radiation dose matter?. Lancet.

[35] Einstein A.J., Henzlova M.J., Rajagopalan S. (2007). Estimating risk of cancer associated with radiation exposure from 64-slice computed tomography coronary angiography. JAMA.

[36] de Jong P.A., Mayo J.R., Golmohammadi K., Nakano Y., Lequin M.H., Tiddens H.A.W.M., Aldrich J., Coxson H.O., Sin D.D. (2006). Estimation of cancer mortality associated with repetitive computed tomography scanning. Am. J. Respir. Crit Care Med..

[37] Brenner D.J., Hall E.J. (2007). Computed Tomography—An Increasing Source of Radiation Exposure. N. Engl. J. Med..

[38] Brenner D.J., Elliston C.D. (2004). Estimated radiation on risks potentially associated with full-body CT screening. Radiology.

[39] White H.A., MacDonald S. (2010). Estimating risk associated with radiation exposure during follow-up after endovascular aortic repair (EVAR). J. Cardiovasc. Surg..

[40] Lehti L., Nyman U., Söderberg M., Björses K., Gottsäter A., Wassélius J. (2016). 80-kVp CT angiography for endovascular aneurysm repair follow-up with halved contrast medium dose and preserved diagnostic quality. Acta Radiol..

[41] Grajo J.R., Sahani D.V. (2018). Dual-Energy CT of the Abdomen and Pelvis: Radiation Dose Considerations. J. Am. Coll. Radiol..

[42] Weinman J.P., Mirsky D.M., Jensen A.M., Stence N.V. (2019). Dual energy head CT to maintain image quality while reducing dose in pediatric patients. Clin. Imaging.

[43] Siegel M.J., Curtis W.A., Ramirez-Giraldo J.C. (2016). Effects of dual-energy technique on radiation exposure and image quality in pediatric body CT. Am. J. Roentgenol..

[44] Goo H.W. (2010). Initial experience of dual-energy lung perfusion CT using a dual-source CT system in children. Pediatr. Radiol..

[45] Primak A.N., Giraldo J.C.R., Eusemann C.D., Schmidt B., Kantor B., Fletcher J.G., McCollough C.H. (2010). Dual-source dual-energy CT with additional tin filtration: Dose and image quality evaluation in phantoms and in vivo. Am. J. Roentgenol..

[46] Macari M., Chandarana H., Schmidt B., Lee J., Lamparello P., Babb J. (2006). Abdominal aortic aneurysm: Can the arterial phase at CT evaluation after endovascular repair be eliminated to reduce radiation dose?. Radiology.

[47] Chandarana H., Godoy M.C.B., Vlahos I., Graser A., Babb J., Leidecker C., Macari M. (2008). Abdominal aorta: Evaluation with dual-source dual-energy multidetector CT after endovascular repair of aneurysms-initial observations. Radiology.

[48] Flors L., Leiva-Salinas C., Norton P.T., Patrie J.T., Hagspiel K.D. (2013). Endoleak detection after endovascular repair of thoracic aortic aneurysm using dual-source dual-energy CT: Suitable scanning protocols and potential radiation dose reduction. Am. J. Roentgenol..

[49] Stolzmann P., Frauenfelder T., Pfammatter T., Peter N., Scheffel H., Lachat M., Schmidt B., Marincek B., Alkadhi H., Schertler T. (2008). Endoleaks after endovascular abdominal aortic aneurysm repair: Detection with dual-energy dual-source CT. Radiology.

[50] Buffa V., Solazzo A., D’auria V., Del Prete A., Vallone A., Luzietti M., Madau M., Grassi R., Miele V. (2014). Dual-source dual-energy CT: Dose reduction after endovascular abdominal aortic aneurysm repair. Radiol. Medica.

[51] Kazimierczak W., Kazimierczak N., Lemanowicz A., Nowak E., Migdalski A., Jawien A., Jankowski T., Serafin Z. (2023). Improved Detection of Endoleaks in Virtual Monoenergetic Images in Dual-Energy CT Angiography Following EVAR. Acad. Radiol..

[52] Iezzi R., Cotroneo A.R., Filippone A., Di Fabio F., Quinto F., Colosimo C., Bonomo L. (2006). Multidetector CT in abdominal aortic aneurysm treated with endovascular repair: Are unenhanced and delayed phase enhanced images effective for endoleak detection?. Radiology.

[53] Glebova N.O., Selvarajah S., Orion K.C., Black J.H., Malas M.B., Perler  B.A., Abularrage  C.J. (2015). Fenestrated endovascular repair of abdominal aortic aneurysms is associated with increased morbidity but comparable mortality with infrarenal endovascular aneurysm repair. J. Vasc. Surg..

[54] Troisi N., Donas K.P., Austermann M., Tessarek J., Umscheid T., Torsello G. (2011). Secondary procedures after aortic aneurysm repair with fenestrated and branched endografts. J. Endovasc. Ther..

[55] Sommer W.H., Becker C.R., Haack M., Rubin G.D., Weidenhagen R., Schwarz F., Nikolaou K., Reiser M.F., Johnson T.R., Clevert D.A. (2012). Time-resolved CT angiography for the detection and classification of endoleaks. Radiology.

[56] Stavropoulos S.W., Charagundla S.R. (2007). Imaging techniques for detection and management of endoleaks after endovascular aortic aneurysm repair. Radiology.

[57] Iezzi R., Cotroneo A.R., Filippone A., Santoro M., Basilico R., Storto M.L. (2008). Multidetector-row computed tomography angiography in abdominal aortic aneurysm treated with endovascular repair: Evaluation of optimal timing of delayed phase imaging for the detection of low-flow endoleaks. J. Comput. Assist. Tomogr..

[58] Javor D., Wressnegger A., Unterhumer S., Kollndorfer K., Nolz R., Beitzke D., Loewe C. (2017). Endoleak detection using single-acquisition split-bolus dual-energy computer tomography (DECT). Eur. Radiol..

[59] Boos J., Fang J., Heidinger B.H., Raptopoulos V., Brook O.R. (2017). Dual energy CT angiography: Pros and cons of dual-energy metal artifact reduction algorithm in patients after endovascular aortic repair. Abdom. Radiol..

[60] Iezzi R., Carchesio F., Posa A., Colosimo C., Bonomo L. (2017). Post-EVAR split-bolus CT angiography using dual-energy CT: All you need in a single scan! In EuroSafe Imaging.

[61] Mehran R., Nikolsky E. (2006). Contrast-induced nephropathy: Definition, epidemiology, and patients at risk. Kidney Int..

[62] van der Molen A.J., Thomsen H.S., Morcos S.K. (2004). Effect of iodinated contrast media on thyroid function in adults. Eur. Radiol..

[63] Katayama H., Yamaguchi K., Kozuka T., Takashima T., Seez P., Matsuura K. (1990). Adverse reactions to ionic and nonionic contrast media. A report from the Japanese Committee on the Safety of Contrast Media. Radiology.

[64] Yamamoto M., Hayashida K., Mouillet G., Hovasse T., Chevalier B., Oguri A., Watanabe Y., Dubois-Randé J.-L., Morice M.-C., Lefèvre T. (2013). Prognostic value of chronic kidney disease after transcatheter aortic valve implantation. J. Am. Coll. Cardiol..

[65] Kane G.C., Doyle B.J., Lerman A., Barsness G.W., Best P.J., Rihal C.S. (2008). Ultra-Low Contrast Volumes Reduce Rates of Contrast-Induced Nephropathy in Patients with Chronic Kidney Disease Undergoing Coronary Angiography. J. Am. Coll. Cardiol..

[66] McDonald R.J., McDonald J.S., Bida J.P., Carter R.E., Fleming C.J., Misra S., Williamson E.E., Kallmes D.F., Paltiel H.J., Gilligan L.A. (2013). Intravenous contrast material-induced nephropathy: Causal or coincident phenomenon?. Radiology.

[67] Huda W., Scalzetti E.M., Levin G. (2000). Technique factors and image quality as functions of patient weight at abdominal CT. Radiology.

[68] van Hamersvelt R.W., Eijsvoogel N.G., Mihl C., de Jong P.A., Schilham A.M.R., Buls N., Das M., Leiner T., Willemink M.J. (2018). Contrast agent concentration optimization in CTA using low tube voltage and dual-energy CT in multiple vendors: A phantom study. Int. J. Cardiovasc. Imaging.

[69] Carrascosa P., Leipsic J.A., Capunay C., Deviggiano A., Vallejos J., Goldsmit A., Rodriguez-Granillo G.A. (2015). Monochromatic image reconstruction by dual energy imaging allows half iodine load computed tomography coronary angiography. Eur. J. Radiol..

[70] Godoy M.C., Heller S.L., Naidich D.P., Assadourian B., Leidecker C., Schmidt B., Vlahos I. (2011). Dual-energy MDCT: Comparison of pulmonary artery enhancement on dedicated CT pulmonary angiography, routine and low contrast volume studies. Eur. J. Radiol..

[71] Nijhof W., Baltussen E., Kant I., Jager G., Slump C., Rutten M. (2016). Low-dose CT angiography of the abdominal aorta and reduced contrast medium volume: Assessment of image quality and radiation dose. Clin. Radiol..

[72] Dubourg B., Caudron J., Lestrat J.-P., Bubenheim M., Lefebvre V., Godin M., Tron C., Eltchaninoff H., Bauer F., Dacher J.-N. (2014). Single-source dual-energy CT angiography with reduced iodine load in patients referred for aortoiliofemoral evaluation before transcatheter aortic valve implantation: Impact on image quality and radiation dose. Eur. Radiol..

[73] Martin S.S., Albrecht M.H., Wichmann J.L., Hüsers K., Scholtz J.-E., Booz C., Bodelle B., Bauer R.W., Metzger S.C., Vogl T.J. (2017). Value of a noise-optimized virtual monoenergetic reconstruction technique in dual-energy CT for planning of transcatheter aortic valve replacement. Eur. Radiol..

[74] Maturen K.E., Kaza R.K., Liu P.S., Quint L.E., Khalatbari S.H., Platt J.F. (2012). “Sweet spot” for endoleak detection: Optimizing contrast to noise using low kev reconstructions from fast-switch kVp dual-energy CT. J. Comput. Assist. Tomogr..

[75] Martin S.S., Wichmann J.L., Weyer H., Scholtz J.-E., Leithner D., Spandorfer A., Bodelle B., Jacobi V., Vogl T.J., Albrecht M.H. (2017). Endoleaks after endovascular aortic aneurysm repair: Improved detection with noise-optimized virtual monoenergetic dual-energy CT. Eur. J. Radiol..

[76] Skawran S., Angst F., Blüthgen C., Eberhard M., Kälin P., Kobe A., Nagy D., Szucs-Farkas Z., Alkadhi H., Euler A. (2020). Dual-Energy Low-keV or Single-Energy Low-kV CT for Endoleak Detection?. Investig. Radiol..

[77] Charalambous S., Perisinakis K., Kontopodis N., Papadakis A.E., Maris T.G., Ioannou C.V., Karantanas A., Tsetis D. (2022). Dual-energy CT angiography in imaging surveillance of endovascular aneurysm repair—Preliminary study results. Eur. J. Radiol..

[78] Ragusi M.A.A.D., van der Meer R.W., Joemai R.M.S., van Schaik J., van Rijswijk C.S.P. (2018). Evaluation of CT Angiography Image Quality Acquired with Single-Energy Metal Artifact Reduction (SEMAR) Algorithm in Patients after Complex Endovascular Aortic Repair. Cardiovasc. Interv. Radiol.

[79] Kazimierczak W., Nowak E., Kazimierczak N., Jankowski T., Jankowska A., Serafin Z. (2023). The value of metal artifact reduction and iterative algorithms in dual energy CT angiography in patients after complex endovascular aortic aneurysm repair. Heliyon.

[80] Albrecht M.H., De Cecco C.N., Nance J.W., Varga-Szemes A., De Santis D., Eid M., Tesche C., Apfaltrer G., Doeberitz P.L.v.K., Jacobs B. (2017). Cardiac Dual-Energy CT Applications and Clinical Impact. Curr. Radiol. Rep..

[81] Mangold S., Cannaó P.M., Schoepf U.J., Wichmann J.L., Canstein C., Fuller S.R., Muscogiuri G., Varga-Szemes A., Nikolaou K., De Cecco C.N. (2016). Impact of an advanced image-based monoenergetic reconstruction algorithm on coronary stent visualization using third generation dual-source dual-energy CT: A phantom study. Eur. Radiol..

[82] Hickethier T., Baeßler B., Kroeger J.R., Doerner J., Pahn G., Maintz D., Michels G., Bunck A.C. (2017). Monoenergetic reconstructions for imaging of coronary artery stents using spectral detector CT: In-vitro experience and comparison to conventional images. J. Cardiovasc. Comput. Tomogr..

[83] De Cecco C.N., Darnell A., Rengo M., Muscogiuri G., Bellini D., Ayuso C., Laghi A. (2012). Dual-energy CT: Oncologic applications. AJR Am. J. Roentgenol..

[84] Leithner D., Wichmann J.L., Vogl T.J., Trommer J., Martin S.S., Scholtz J.-E., Bodelle B., De Cecco C.N., Duguay T., Nance J.W. (2017). Virtual Monoenergetic Imaging and Iodine Perfusion Maps Improve Diagnostic Accuracy of Dual-Energy Computed Tomography Pulmonary Angiography with Suboptimal Contrast Attenuation. Investig. Radiol..

[85] Borges A.P., Antunes C., Curvo-Semedo L. (2023). Pros and Cons of Dual-Energy CT Systems: “One Does Not Fit All”. Tomography.

[86] Goo H.W., Goo J.M. (2017). Dual-energy CT: New horizon in medical imaging. Korean J. Radiol..

[87] Katsura M., Sato J., Akahane M., Kunimatsu A., Abe O. (2018). Current and novel techniques for metal artifact reduction at CT: Practical guide for radiologists. Radiographics.

[88] Forghani R., De Man B., Gupta R. (2017). Dual-Energy Computed Tomography: Physical Principles, Approaches to Scanning, Usage, and Implementation: Part 2. Neuroimaging Clin. N. Am..

[89] Toia G.V., Mileto A., Wang C.L., Sahani D.V. (2022). Quantitative dual-energy CT techniques in the abdomen. Abdom. Radiol..

[90] Parakh A., An C., Lennartz S., Rajiah P., Yeh B.M., Simeone F.J., Sahani D.V., Kambadakone A.R. (2021). Recognizing and minimizing artifacts at dual-energy CT. Radiographics.

[91] Petritsch B., Pannenbecker P., Weng A.M., Grunz J.-P., Veldhoen S., Bley T.A., Kosmala A. (2021). Split-filter dual-energy CT pulmonary angiography for the diagnosis of acute pulmonary embolism: A study on image quality and radiation dose. Quant. Imaging Med. Surg..

[92] Wortman J.R., Sodickson A.D. (2018). Pearls, Pitfalls, and Problems in Dual-Energy Computed Tomography Imaging of the Body. Radiol. Clin. N. Am..

[93] Wichmann J.L., Hardie A.D., Schoepf U.J., Felmly L.M., Perry J.D., Varga-Szemes A., Mangold S., Caruso D., Canstein C., Vogl T.J. (2017). Single- and dual-energy CT of the abdomen: Comparison of radiation dose and image quality of 2nd and 3rd generation dual-source CT. Eur. Radiol..

[94] Schmidt D., Söderberg M., Nilsson M., Lindvall H., Christoffersen C., Leander P. (2018). Evaluation of image quality and radiation dose of abdominal dual-energy CT. Acta Radiol..

[95] Harder A.M.D., Willemink M.J., de Ruiter Q.M., Schilham A.M., Krestin G.P., Leiner T., de Jong P.A., Budde R.P. (2015). Achievable dose reduction using iterative reconstruction for chest computed tomography: A systematic review. Eur. J. Radiol..

[96] Parakh A., Macri F., Sahani D. (2018). Dual-Energy Computed Tomography: Dose Reduction, Series Reduction, and Contrast Load Reduction in Dual-Energy Computed Tomography. Radiol. Clin. N. Am..

[97] Mohammadinejad P., Mileto A., Yu L., Leng S., Guimaraes L.S., Missert A.D., Jensen C.T., Gong H., McCollough C.H., Fletcher J.G. (2021). Ct noise-reduction methods for lower-dose scanning: Strengths and weaknesses of iterative reconstruction algorithms and new techniques. Radiographics.

[98] Dunet V., Bernasconi M., Hajdu S.D., Meuli R.A., Daniel R.T., Zerlauth J.-B. (2017). Impact of metal artifact reduction software on image quality of gemstone spectral imaging dual-energy cerebral CT angiography after intracranial aneurysm clipping. Neuroradiology.

